# An evaluation of the psychometric properties of the Indicator of Relative Need (IoRN) instrument

**DOI:** 10.1186/s12877-016-0321-3

**Published:** 2016-07-28

**Authors:** Anne Canny, Frances Robertson, Peter Knight, Adam Redpath, Miles D. Witham

**Affiliations:** 1 Ageing and Health, University of Dundee, Dundee, UK; 2 Joint Improvement Team, Scottish Government, ᅟ, ᅟ; 3 Ageing and Health, Ninewells Hospital, Dundee, DD1 9SY UK

**Keywords:** Older people, Informatics, Health care, Social care, Integrated care, Function, Dependency, Assessment, Rehabilitation, Care planning

## Abstract

**Background:**

The Indicator of Relative Need (IoRN) instrument is designed for both health and social care services to measure function and dependency in older people. To date, the tool has not undergone assessment of validity. We report two studies aimed to evaluate psychometric properties of the IoRN.

**Methods:**

The first study recruited patients receiving social care at discharge from hospital, those rehabilitating in intermediate care, and those in a rehabilitation at home service. Participants were assessed using the IoRN by a single researcher and by the clinical team at baseline and 8 weeks. Comparator instruments (Barthel ADL, Nottingham Extended ADL and Townsend Disability Scale) were also administered. Overall change in ability was assessed with a 7 point Likert scale at 8 weeks. The second study analysed linked routinely collected, health and social care data (including IoRN scores) to assess the relationship between IoRN category and death, hospitalisation and care home admission as a test of external validity.

**Results:**

Ninety participants were included in the first study, mean age 77.9 (SD 12.0). Cronbach’s alpha for IoRN subscales was high (0.87 to 0.93); subscales showed moderate correlation with comparator tools (*r* = 0.43 to 0.63). Cohen’s weighted kappa showed moderate agreement between researcher and clinician IoRN category (0.49 to 0.53). Two-way intraclass correlation coefficients for IoRN subscales in participants reporting no change in ability were high (0.88 to 0.98) suggesting good stability; responsiveness coefficients in participants reporting overall change were equal to or better than comparator tools. 1712 patients were included in the second study, mean age 81.0 years (SD 7.7). Adjusted hazard ratios for death, care home admission and hospitalisation in the most dependent category compared to the least dependent IoRN category were 5.9 (95 % CI 2.0–17.0); 7.2 (95 % CI 4.4–12.0); 1.1 (95 % CI 0.5–2.6) respectively. The mean number of allocated hours of care 6 months after assessment was higher in the most dependent group compared to the least dependent group (5.6 vs 1.4 h, *p* = 0.005).

**Conclusions:**

Findings from these analyses support the use of the IoRN across a range of clinical environments although some limitations are highlighted.

## Background

The numbers of people with complex health conditions are increasing globally [1]. The United Kingdom (UK) is no exception to this growth, meaning that demands for health and social care systems are also rising [2]. In response, the UK Government has made health and social care integration a priority to improve outcomes for people who use their services and to maximize finite resources [3]. Joined-up services are required to co-ordinate person-centred and holistic care needs across more than one agency or discipline [4]. However, for health and social care integration to prove successful, shared knowledge, resources, and learning are required [5]. One way to help facilitate this is through shared assessment tools.

The Indicator of Relative Need (IoRN) was originally developed in Scotland by the Scottish Executive and the Information Services Division of the Scottish Government with the active involvement of social work teams and is already in widespread use by many health and social care teams. The tool was originally developed to classify older people into groups according to their level of dependency in order to inform decisions on the provision of care packages appropriate to their need. The IoRN questionnaire is administered by a social worker or other healthcare professional when conducting a full adult assessment. The tool is divided into four main domains to measure ability to mobilise, ability to attend to personal care, mental health, and bowel care management. Scores from IoRN are combined via an algorithm into a final category ranging from A1 to I, with A1 being the least dependent and I the most dependent category (http://www.jitscotland.org.uk/action-areas/data/iorn/) [6].

IoRN has since been further applied to support the Single Shared Assessment (SSA) process or its local equivalent, which addresses health and social care need of all adults, not just older people. In addition, the IoRN has recently been piloted for a range of additional purposes, as a tool to measure improvement after reablement or rehabilitation. A different version of the IoRN is available for use in care homes as an index of dependency and to support a staffing model [7].

A key obstacle to the use of the IoRN across health and social care services is the lack of detailed validation data. The psychometric properties of the IoRN tool have not been subjected to independent scrutiny to date. Such data are important to ensure that the tool is capable of measuring what it claims to measure, that the measurements are reliable, and that if improvements or deterioration in function are produced, the tool is capable of detecting these.

In order for agencies to be able to adopt the IoRN with confidence, the instrument requires rigorous examination to provide evidence of suitability whilst also making clear any limitations and boundaries to its appropriate use. This study therefore aimed to evaluate the psychometric properties of the IoRN: 1) inter-rater variability; 2) reliability; 3) responsiveness to change; 4) external (criterion) validity.

## Methods

Two studies are reported in this paper. Study 1 was a prospective analyses involving a range of clients from different settings who provided sociodemographic details alongside information from the IoRN instrument and other comparable tools, [Barthel Activities of Daily Living (ADL) Index; Nottingham Extended ADL (NEADL); Townsend Disability Scale (TDS)] [8–10]. Study 1 addressed the first 3 aims; inter- rater variability, reliability and responsiveness to change in the IoRN. Study 2 was retrospective in nature and analysed linked routinely collected health and social care data including IoRN assessments. Study 2 focused on the fourth objective: external validity.

### Study 1 (prospective)

#### Participants

Participants aged 18 and over from NHS Tayside were recruited to the study. Clients who were admitted to an intermediate care unit, care at home scheme, or discharged from hospital with a Dundee City Council care package were invited to participate. A single research nurse liaised with participating units and provided potential participants with information sheets and consent forms in advance for their consideration. Mutually convenient appointments were arranged by the research nurse with participants who expressed an interest in taking part. Participants unable to give written informed consent, those who previously participated in the study (i.e. during a previous rehabilitation admission), and those who were not expected to live to discharge from units were excluded from the study.

#### Data collection

A single research nurse gathered socio-demographic information from each participant once written informed consent was obtained i.e. age, gender, number of medications, medication dispensing aids, meals on wheels, living arrangements, district nurse use, informal care, package of care.

The following assessment tools were administered both at the beginning of the study and after an 8 week follow-up period.

1. IoRN scores were completed independently by the research nurse and the clinical team. Both measures were obtained by using information already recorded in medical, nursing and social work notes. A comparison of how the researcher and the clinical team interpreted the same data to score the IoRN was made in order to test inter-rater variability.
2. The 10 point Barthel Index [11] measured basic activities of daily living as a comparator to IoRN and was calculated by the research nurse based on information obtained from clinical notes.
3. Nottingham Extended ADL Scale (NEADL) [12, 13] and Townsend Disability Scale (TDS) [14, 15] were administered to each participant on a one-to-one basis by the research nurse as supplementary comparators to IoRN.

Overall change in function between baseline and follow-up was measured using a 7 point Likert scale ranging from 1 to 7, with higher scores indicating greater improvement, i.e. much worse; worse; a little worse; no change; a little better; better; much better. This was completed by the participant, and an additional Likert measure was completed by the care team. Data from all instruments were entered and analysed using SPSS v21. A 2 sided *p* value of <0.05 was classed as significant for all analyses.

#### Analyses

Cronbach’s alpha was calculated to measure internal consistency between IoRN subscales from the research nurse and care teams over the three different settings. Construct validity was calculated by performing Pearson’s correlation with IoRN subscores and other measures of function (Barthel, NEADL, Townsend and mental health scores) alongside intensity of care package. Cohen’s Kappa (weighted) examined associations between IoRN subscores calculated by the research nurse and the clinical team to test Inter-rater variability.

Cohen’s d (effect size) and Guyatt’s responsiveness coefficient were used to test responsiveness to change [16, 17]. For Cohen’s d, all the ‘worse’ categories on the Likert scale were aggregated due to small numbers. Similarly all of the improvement categories were aggregated into a single ‘better’ category. Cohen’s d was calculated as: *(Mean difference between baseline and follow up) / (pooled SD of baseline and follow up*. Where pooled SD was calculated as the square root of: *((baseline n-1 × SD baseline) + (followup n-1 × SD followup)) / (baseline n + followup n)*.

Guyatt’s responsiveness coefficient was calculated using the minimum clinically important difference (MCID), taken as those noting either slight improvement or slight worsening on the Likert scale. The coefficient is calculated as; mean change in score in group showing slight improvement or slight worsening on Likert / SD of change score in group showing ‘no change’ on Likert.

### Study 2 (retrospective)

#### Participants

Routinely collected health and social care data from NHS Tayside and Dundee City Council were linked and analysed through the Health Informatics Centre (HIC) Safe Haven at the University of Dundee. Databases were probabilistically linked using name, date of birth and postcode alongside clerical review procedures to obtain >95 % accuracy of patient matching [18]. Clients who had been assessed with IoRN by Dundee Social Work Department between 2008 and 2012 were selected from the matched data. Details of the linked data sets used in this analysis has been published previously [19].

#### Data collection and analyses

Analyses examined external validity of the IoRN by testing associations between IoRN categories and outcomes that would be expected to be related to IoRN score, namely death, hospitalization and care home admission. Associations between IoRN scores and time to death, time to hospital admission and time to care home admission using Cox regression models were performed. Models were adjusted for age, gender, number and length of stays in hospital in the previous year; all factors known to be important predictors of death and rehospitalisation and used in other scores e.g. Scottish Patients at Risk of Readmission and Admission (SPARRA) [20]. IoRN subscores were entered into Cox regression analyses (forced entry) without other adjustment.

Care allocation data (number of hours of home care allocated per person per week) recorded by Dundee Social Work Department were also examined in association with IoRN scores. Student’s *t*-test was performed to compare the mean weekly hours of allocated care at 6 months after the IoRN assessment, excluding clients who had died or been admitted to a care home. All analyses were performed using SPSS version 21. A 2 sided *p* value of <0.05 was taken as significant for all analyses.

## Results

### Study 1 - prospective

#### Baseline study details

Ninety participants were recruited between October 2014 and September 2015. Table 1 shows baseline details. Group A comprised those admitted to rehabilitation or intermediate care; group B comprised those admitted to an intermediate care at home scheme, and Group C comprised those discharged from hospital with a package of care.

**Table 1:** Study 1 baseline details by group

	Group A (*n* = 41)	Group B (*n* = 27)	Group C (*n* = 22)	All (*n* = 90)
Mean age (SD)	79.4 (12.1)	74.0 (13.8)	80.2 (7.9)	77.9 (12.0)
Female sex (%)	33 (80)	20 (74)	14 (64)	67 (74)
Mean no. of medications (SD)	7.1 (4.4)	7.2 (4.1)	7.2 (2.9)	7.1 (4.0)
Use of medication dispensing aid (%)	8 (20)	7 (26)	5 (23)	20 (22)
Meals on wheels (%)	4 (10)	4 (15)	4 (18)	12 (13)
District nurse assistance (%)	4 (10)	5 (19)	5 (23)	14 (16)
Lives alone (%)	30 (73)	18 (67)	12 (55)	60 (67)
Informal carer (%)	35 (85)	25 (93)	22 (100)	82 (91)
Package of care planned (%)	21 (51)	13 (48)	22 (100)	56 (62)
Mean Barthel score (SD)	15.0 (3.3)	15.3 (5.0)	13.7 (4.5)	14.8 (4.2)
Mean NEADL score (SD)	33 (13)	34 (13)	39 (12)	35 (13)
Mean TDS score (SD)	13.0 (2.9)	12.3 (4.0)	13.9 (3.5)	13.0 (3.4)
IoRN ADL score (SD)	3.6 (0.9)	3.9 (2.5)	4.6 (1.6)	3.9 (1.7)
IoRN personal care score (SD)	18.4 (6.7)	17.2 (8.4)	22.9 (7.1)	19.1 (7.6)
IoRN mental health score) (SD)	6 (−) ^a^	6 (−) ^a^	6.0 (0.2)	6.0 (0.1)
IoRN high bowel care (%)	4 (10)	1 (4)	5 (23)	10 (11)

^a^all scores identical therefore SD not computable. All IoRN scores from researcher completed tools

#### Questionnaire completion rates

**Fig 1:**
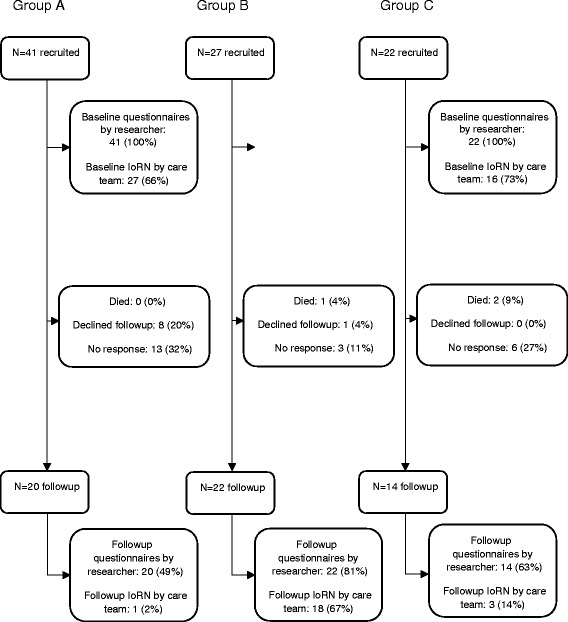
Flowchart showing completion rates for questionnaires

Completion rates for questionnaires varied considerably between baseline and follow-up for the research nurse and care teams (Fig. 1). The analysis revealed overall completion rates at baseline and follow-up respectively: Research Nurse IoRN (100 and 61 %); Barthel/TDS/NEADL (100 and 62 %), Care Teams IoRN (72 and 24 %). There was also a marked difference in completion rates between group care teams at baseline and follow-up respectively: Group A (66 and 2 %); Group B (81 and 67 %); and Group C (73 and 14 %). From 90 participants, 3 % died before follow up (*n* = 3) and 10 % declined follow-up (*n* = 9).

#### Internal consistency

Overall Cronbach’s alpha for ADL and personal care subscales from the research nurse and care teams indicated a high degree of internal consistency. IoRN ADL and personal care for the research nurse (0.87 and 0.92) respectively, IoRN ADL and personal care for care teams (0.93 and 0.88) respectively. Mental health scores were almost universally normal, therefore lacked sufficient variation to calculate Cronbach’s alpha. These results are consistent with Cronbach’s alpha from stage 2 (retrospective); IoRN ADL (0.80); IoRN personal care (0.91); IoRN mental health (0.71).

#### Construct validity

Table 2 shows how IoRN subscales relate to two measures of activities of daily living (Barthel and NEADL) and one measure of disability (TDS). The IoRN ADL and personal care scores show moderate correlations with these measures, as well as with the intensity of planned care package at the time of assessment, suggesting that these IoRN scales are measuring a related construct (convergent validity). The IoRN mental health score is only weakly correlated with the other scores – this is again to be expected as this is a very different construct, suggesting that this subscale has discriminant validity.

**Table 2:** Correlations between IoRN sub-scores and other measures (baseline IoRN data collected by research nurse)

	IoRN ADL score		IoRN Personal care score		IoRN mental health score	
	*r*	*p*	*r*	*p*	*r*	*p*
Barthel score	−0.55	<0.001	−0.54	<0.001	0.13	0.21
NEADL score total	0.52	<0.001	0.63	<0.001	−0.16	0.13
TDS score	0.43	<0.001	0.62	<0.001	−0.16	0.15
NEADL mobility	0.31	0.003	0.38	<0.001	−0.23	0.03
NEADL kitchen	0.48	<0.001	0.58	<0.001	−0.09	0.40
NEADL domestic	0.41	<0.001	0.50	<0.001	−0.11	0.31
NEADL leisure	0.42	<0.001	0.50	<0.001	−0.05	0.65
Intensity of care package^a^	0.47	<0.001	0.39	<0.001	0.05	0.65

Pearson’s correlation exc for ^a^Spearman’s rho (categorisation of care: 1×/week; 1×/day; 2×/day; 4×/day)

#### Inter-rater variability

Cohen’s kappa (weighted) was used to quantify inter-rater variability in IoRN categorisation. When treating each subcategory within A and B as separate categories (i.e. A1, A2, A3 all separate), weighted kappa was 0.49. If subcategories were aggregated (i.e. treating A1, A2, A3 all as category A), kappa was 0.53. These values suggest moderate agreement between raters. Agreement between the researcher and the clinical team was much better for groups B and C than it was for group A. Excluding group A yielded somewhat better kappa values of 0.58 and 0.64.

#### Change in scores over time

To analyse whether changes in IoRN scores correlated with changes in overall function, we asked both the clinical team and the client how their overall function had changed between baseline and follow up, using a seven-point Likert scale. This dual approach was used as the perceptions of the client may differ from the perceptions of the care team. Changes in both IoRN subscales and changes in Barthel, NEADL and TDS scores are shown subdivided by Likert category from the care team and from the client (Table 3). Inter- rater agreement between the care team and client Likert categories (weighted kappa) was moderate at 0.64 (*p* < 0.001).

**Table 3:** Likert categories from care team (overall change)

	Much worse *N* = 0	Worse *N* = 3	Slightly worse *N* = 1	No change *N* = 13	Slightly better *N* = 25	Better *N* = 8	Much better *N* = 6	R (P for trend) ^a^ *N* = 56
Barthel difference (SD)	–	−8.0 (9.2)	−5.0 (−)	0.5 (1.2)	1.5 (3.2)	1.9 (1.8)	0.7 (2.0)	0.31 (0.02)
NEADL difference (SD)	–	27 (12)	11 (−)	1 (10)	−1 (10)	−7 (10)	−7 (15)	−0.39 (0.003)
TDS difference (SD)	–	4.7 (0.6)	1 (−)	0.9 (2.0)	0.8 (2.5)	−1.4 (2.9)	−3.4 (5.3)	−0.44 (0.001)
IoRN ADL difference (SD)	–	4.7 (0.6)	0.0 (−)	0.1 (0.7)	−0.7 (2.0)	−0.5 (0.8)	0.2 (0.4)	−0.26 (0.05)
IoRN personal care difference (SD)	–	18.0 (4.4)	3.0 (−)	−0.6 (4.7)	−2.8 (4.9)	−4.4 (4.7)	−5.7 (6.1)	−0.51 (<0.001)
IoRN category change	–	3/3 worse category	0/1 worse category	10/13 same category	10/25 better category	6/8 better category	3/6 better category	–

^a^Spearman’s rho

These categorisations then formed the basis for assessing the reliability (stability) of each score in clients who selected ‘no change’ on the Likert scale, and assessing the responsiveness to change for those clients perceiving that they had improved or worsened.

#### Reliability

To estimate reliability (also known as stability), the change in scores between baseline and follow up for those reporting ‘no change’ on the overall Likert scales (i.e. those who remained stable in perceived function) were compared using intra-class correlation coefficients (ICC). Results are given in Table 4. The ICC values for both the ADL and personal care subscales were very high, suggesting a high degree of stability of these measures in stable clients. The mental health subscale lacked sufficient variability to allow testing.

**Table 4:** Intra-class correlation coefficients for those reporting ‘no change’ on Likert scale

	Team assessment		Client assessment	
	ICC	P	ICC	P
Barthel	0.98	<0.001	0.99	<0.001
NEADL	0.83	<0.001	0.76	<0.001
TDS	0.82	<0.001	0.54	0.015
IoRN ADL	0.96	<0.001	0.98	<0.001
IoRN personal care	0.88	<0.001	0.97	<0.001

(2 way, random effects, for absolute agreement)

#### Responsiveness

Two indices of responsiveness were calculated – Cohen’s d (effect size) and Guyatt’s responsiveness coefficient. Results are shown in Table 5.

**Table 5:** Cohen’s d and Guyatt’s responsiveness coefficient

	Worse team opinion (*n* = 4) ^a^	Worse client opinion (*n* = 7) ^a^	Better team opinion (*n* = 39) ^a^	Better client opinion (*n* = 35) ^a^	Slightly worse team opinion (*n* = 1)†	Slightly worse client opinion (*n* = 5)†	A little better team opinion (*n* = 25)†	A little better client opinion (*n* = 22)†
Barthel	0.38	0.18	0.11	0.15	NC	2	1.25	2.5
NEADL	0.3	0.05	0.07	0.06	NC	0.67	0.1	0.44
TDS	3.95	0.17	0.2	0.18	NC	0.69	0.4	0.08
IoRN ADL	1.17	0.77	0.77	0.62	NC	3.5	1	2.25
IoRN personal care	0.24	0.16	0.1	0.09	NC	2.57	0.6	1.62

*NC* Not calculable. ^a^Cohen’s D †Guyatt’s responsiveness

These results suggest that the IoRN scores are no less responsive than the Barthel, NEADL or TDS scores, and indeed the IoRN ADL subscale showed considerably better responsiveness on both the Guyatt’s and Cohen’s measures. Almost all of the scores appeared more responsive to worsening than to improvement.

### Study 2 - retrospective study

#### Baseline study details

A total of 1712 patients were included in the analyses. Mean age was 81.0 years (SD 7.7), and 1200 (70 %) were female. 142 (8 %) died during follow up. Cox proportional hazard regression analyses for time to death, care home admissions and next unscheduled hospital admission are shown in Table 6.

**Table 6:** Cox regression analysis for time to death, care home, hospital admission, and hours of care per week at 30 days post-assessment, adjusted for age, gender, number and length of stays in hospital in the previous year

IoRN Category	Death		Care Home Admission		Hospital Admission		Hours of care per week 30 Days	
	HR	95 % CI	HR	95 % CI	HR	95 % CI	Mean hours	95 % CI
A1	1	–	1	–	1	–	1.39	(0.45–2.34)
A2	2.03	0.57–7.21	0.57	0.28–1.16	0.42	0.17–1.03	2.14	(0.80–3.47)
A3	2.07	0.67–6.35	0.88	0.52–1.46	0.37	0.17–0.81	1.63	(0.94–2.32)
B1	1.50	0.42–5.30	1.36	0.80–2.31	1.08	0.50–2.31	1.97	(1.07–2.88)
B2	2.30	0.74–7.02	1.16	0.68–1.96	1.12	0.51–2.47	2.00	(1.15–2.84)
B3	2.01	0.59–6.91	1.08	0.62–1.89	0.65	0.29–1.41	2.52	(1.02–4.02)
C	4.17	1.41–12.32	1.37	0.83–2.25	0.58	0.25–1.34	2.35	(1.27–3.43)
D	3.20	1.05–9.74	1.29	0.74–2.25	1.36	0.59–3.14	1.35	(0.47–2.22)
E	1.70	0.45–6.24	2.71	1.62–4.54	2.18	0.94–5.08	1.75	(0.59–2.91)
F	4.30	9.50–19.05	3.72	1.88–7.34	2.09	0.26–16.94	2.09	(−1.49–5.68)
G	3.54	1.17–10.66	2.82	1.70–4.69	0.89	0.44–1.79	2.60	(0.59–4.62)
H	5.04	1.46–17.40	8.61	4.94–15.04	1.25	0.38–4.11	0.00	(−)
I	5.88	2.04–16.97	7.22	4.35–11.97	1.15	0.51–2.59	5.55	(1.86–9.24)

The mean number of hours of care received per week was compared between IoRN categories. The only significant finding occurred between the least dependent category (A1) and the most dependent category I. A1 (mean = 1.4 h, 95 % CI 0.5–2.3 h) and I (mean = 5.6 h, 95 % CI 1.9–9.2 h) p <0.05 by ANOVA.

To test the contribution that each individual sub-score from the IoRN made to prediction of death hospitalisation or care home admission, all sub-scores were entered into Cox regression analyses (forced entry) without other adjustment. Results are shown in Table 7.

**Table 7:** Using sub-scores from IoRN to predict death, hospitalisation or care home admission

	HR Death (95 % CI)	HR Hospitalisation (95 % CI)	HR Care home admission (95 % CI)
Bowel function - high vs low	2.00 (1.35–2.94)	0.84 (0.51–1.38)	2.37 (1.91–2.94)
ADL - per point	1.18 (1.11–1.26)	0.99 (0.90–1.09)	1.23 (1.19–1.26)
Personal care - per point	1.04 (1.02–1.06)	0.99 (0.87–1.01)	1.08 (1.07–1.09)
Mental health - per point	1.10 (1.01–1.20)	0.89 (0.82–0.95)	1.29 (1.24–1.35)

## Discussion

The results show IoRN performed well on a range of psychometric measures. Internal consistency was good, subscales correlated with comparable instruments, and did not show any association with unrelated tools (e.g. mental health) demonstrating convergent and discriminant validity. IoRN demonstrated stability in clients whose overall function did not change. Responsiveness to change was not high, however it was no worse than the performance of other commonly used measures of disability and function in this cohort. Responsiveness to deterioration appeared to be stronger than for improvement. Inter-rater reliability was good for subscale scores, but only moderate for Individual IoRN category scores. Performance seemed to be highly dependent upon training and engagement of clinical teams. The importance of consistent training of teams cannot be emphasized enough as well as taking note that attempts to impose the IoRN on reluctant teams are likely to generate poor quality data.

External validity was demonstrated indicating IoRN as a very good predictor of future care home admission, a good predictor of death, but IoRN did not predict future hospital admission in this analysis. The lack of association between IoRN category and risk of future hospital admission is likely to reflect that there are multiple other factors that impact on the risk of hospital admission (e.g. undiagnosed conditions, frailty, medication adherence, body mass index), which the current analysis could not measure. The IoRN was not explicitly designed to predict hospitalisation and so it is perhaps unsurprising that it does not do so. It is also possible that those in higher IoRN categories receive more care which may have reduced the risk of future hospitalization, thus weakening any association. Similarly, incorporating previous hospitalisations into the predictive model is likely to have weakened any added predictive value that the IoRN might have for future hospitalisation.

The IoRN tool has not undergone published psychometric assessment before, and there is thus no existing literature with which to compare our results. There are many tools in use within healthcare that have been validated for assessment of impairment, disability, daily function and care needs; how then does the IoRN tool fit into this already crowded landscape?

Assessment tools can be divided into 1st, 2nd and 3rd generation instruments that measure function and dependency in older people. 1st generation tools (of which IoRN is one) focus within a single or limited set of domains, for example, Barthel activities of daily living, Nottingham Extended ADL Scale and Townsend disability scale [8, 12, 14]. 2nd generation tools gather information from multidimensional fields for instance, Easy Care health assessments [21], and FACE (functional analyses of care environments) [22]. 3rd generations extend 2nd generation models by applying supporting software to fully integrate collections of measurements to enable transfer of information across different health and social care settings, as exemplified by the Inter-RAI suite [23].

Implementing 2nd and 3rd generation instruments into current health and social care systems, at least in the UK, presents significant challenges. For example, a series of case studies from 8 countries illustrated the difficulties associated with employing Inter-RAI across different health and social systems and cultures who hold conflicting approaches to gathering client information [24]. This was particularly relevant to the UK where reports of ‘too lengthy’ and ‘too clinical’ were recorded [24]. Little flexibility was provided to incorporate free-text information to capture important nuanced and contextual information making results difficult to interpret [24]. Thus although the IoRN tool captures data around a more limited set of domains, it has been designed for use by social care practitioners, and sits within existing assessment processes. Social care practitioners may be more likely to accept the IoRN because they may be unfamiliar with many of the tools already used in healthcare assessment. Conversely, although healthcare practitioners already have many tools to choose from, the results of this assessment show that IoRN is a viable alternative tool for many assessment uses within healthcare - and support its use as a tool acceptable to both health and social care practitioners [25]. Whilst the comprehensive, consistent approach across multiple care settings of second and third generation tools are advantages, such benefits cannot be realised if practitioners are unwilling to use such tools; in these circumstances, a less-perfect first generation tool in widespread use is preferable to a more sophisticated tool that is not used. The widespread uptake of the IoRN within Scotland suggests that such first generation tools are perceived as having advantages by both health and social care practitioners.

### Limitations to study

A number of limitations to the studies merit discussion. Completion rates for the IoRN by some clinical teams in the prospective study were not as high as anticipated, and in the case of one team, significant concerns existed about the quality and completeness of the IoRN tool data collection by the clinical team. This was despite standardized training given to all participating teams prior to the start of data collection. Although we cannot provide empirical data to give insight into why this might be, levels of engagement with the IoRN and with the project varied across teams, and was noticeably weaker in the group with poor results. Staff in this group were a mixture of public-sector healthcare staff and private-sector care home staff, with reactive, intermittent input from senior medical staff. This group was also characterised by short length of stay, rapid turnover of patients and high bed occupancy. It is therefore possible that the low quality of data collected in this group is due to a combination of a high-pressure care environment where the emphasis is on discharge, together with training, cultural and leadership factors affecting performance of the team. These findings serve as a reminder that simply providing training and resources to teams is not sufficient to ensure high-quality data collection, even with relatively simple tools.

A further issue limiting the power of the prospective study was the relatively low rate of successful follow- up. For some teams, this was due to short stays in the intermediate care unit, without a clear mechanism for follow-up in routine clinical care. This was a particular problem for patients enrolled at the point of hospital discharge with a package of care. For example, hospitals involved did not allocate a designated social worker to individuals when returning patients to the community. Tracing patients through social work was difficult and even when they were located, social workers, who incidentally were not affiliated to the study, and already under considerable work pressure, did not see an IoRN assessment as a priority.

Numbers with successful follow-up by the researcher were higher, but even here a combination of deaths, illness, client reluctance and an inability to contact clients at their usual address contributed to attrition of follow-up. Whilst this led to some loss of power for the prospective study, it did not prevent the project from being able to draw conclusions. Our inability to analyse Mental Health sub-scores was as a result of limited range in the results. Almost all participants scored the lowest possible mental health score. This is difficult to avoid in a prospective study requiring client capacity to consent. Clients who would score high on the mental health sub-scores would likely hold significant cognitive impairment which would prevent them from taking part in the study.

The retrospective study used routinely collected data from clients aged 65 years and over as this was standard practice of the time. Whereas the prospective study IoRN analysed data with clients aged 18 years and over. Mean ages between the two studies however scored similar (81 versus 79.9 years respectively) and were unlikely to affect findings. Finally, data on the relationship between IoRN category and care needs are limited by the fact that the social work dataset used for this analysis captured formal, but not informal care (i.e. care by relatives, friends). This is likely to have weakened any relationship between IoRN category and the amount of care provided.

## Conclusions

Findings from these analyses support the use of the IoRN across a range of clinical environments. Some caveats around different uses of the IoRN are worthy of note. IoRN is less responsive to improvement but has a better responsiveness to deterioration and may be useful for detecting this. IoRN is suitable for predicting mortality, future care home admission, and future need for care home provision at service level. It is unlikely to be useful to predict risk of future hospitalization. Alternative tools (e.g. Scottish Patients at Risk of Readmission and Admission – SPARRA) exist to examine this risk.

## Abbreviations

ADL, activities of daily living; HIC, Health Informatics Centre; IoRN, indicator of relative need; MCID, minimum clinically important difference; NEADL, Nottingham extended activities of daily living; SPARRA, Scottish patients at risk of readmission and admission; SSA, single shared assessment; TDS, Townsend Disability Scale; UK, United Kingdom; UN, United Nations; WHO, World Health Organisation.
